# Alterations in blood proteins in the prodromal stage of bipolar II disorders

**DOI:** 10.1038/s41598-022-07160-0

**Published:** 2022-02-24

**Authors:** Hyunju Lee, Dohyun Han, Sang Jin Rhee, Jayoun Kim, Yunna Lee, Eun Young Kim, Dong Yeon Park, Sungwon Roh, Myungjae Baik, Hee Yeon Jung, Junhee Lee, Tae Young Lee, Minah Kim, Hyunsuk Shin, Hyeyoon Kim, Se Hyun Kim, Jun Soo Kwon, Yong Min Ahn, Kyooseob Ha

**Affiliations:** 1 Department of Neuropsychiatry, Seoul National University Hospital, 101, Daehak-ro, Jongno-gu, Seoul, 03080 Republic of Korea; 2 Department of Psychiatry and Behavioral Science, Seoul National University College of Medicine, Seoul, Republic of Korea; 3 Proteomics Core Facility, Biomedical Research Institute, Seoul National University Hospital, Seoul, Republic of Korea; 4 Transdisciplinary Department of Medicine and Advanced Technology, Seoul National University Hospital, Seoul, Republic of Korea; 5 Medical Research Collaborating Center, Seoul National University Hospital, Seoul, Republic of Korea; 6 Department of Neuropsychiatry, Kosin University Gospel Hospital, Busan, Republic of Korea; 7 Department of Medicine, Seoul National University College of Medicine, Seoul, Republic of Korea; 8 Department of Psychiatry, National Center for Mental Health, Seoul, Republic of Korea; 9 Department of Neuropsychiatry, Hanyang University Hospital, Seoul, Republic of Korea; 10 Department of Psychiatry, Kyung Hee University Medical Center, Kyung Hee University School of Medicine, Seoul, Republic of Korea; 11 Department of Psychiatry, SMG-SNU Boramae Medical Center, Seoul, Republic of Korea; 12 Department of Psychiatry, Uijeongbu Eulji Medical Center, Uijeongbu, Republic of Korea; 13 Department of Neuropsychiatry, Pusan National University Yangsan Hospital, Yangsan, Republic of Korea; 14 Department of Pathology, Seoul National University College of Medicine, Seoul, Republic of Korea; 15 Institute of Human Behavioral Medicine, Seoul National University Medical Research Center, Seoul, Republic of Korea

**Keywords:** Biochemistry, Biomarkers, Psychiatric disorders

## Abstract

Although early intervention may help prevent the progression of bipolar disorder, there are some controversies over early pharmacological intervention. In this study, we recruited 40 subjects in the prodromal stage of BD-II (BP), according to bipolar at-risk state criteria. We compared the expression of their plasma proteins with that of 48 BD-II and 75 healthy control (HC) to identify markers that could be detected in a high-risk state. The multiple reaction monitoring method was used to measure target peptide levels with high accuracy. A total of 26 significant peptides were identified through analysis of variance with multiple comparisons, of which 19 were differentially expressed in the BP group when compared to the BD-II and HC groups. Two proteins were overexpressed in the BP group; and were related to pro-inflammation and impaired neurotransmission. The other under-expressed peptides in the BP group were related to blood coagulation, immune reactions, lipid metabolism, and the synaptic plasticity. In this study, significant markers observed in the BP group have been reported in patients with psychiatric disorders. Overall, the results suggest that the pathophysiological changes included in BD-II had already occurred with BP, thus justifying early pharmacological treatment to prevent disease progression.

## Introduction

Bipolar disorders (BDs) pose a higher risk of functional impairment or irreversible change as their courses become chronic. In particular, the suicide rate with BD is approximately 10 to 30 times higher than that of the general population^1^. Despite continuous advancements in neuroscience, predicting these problematic behaviors is still challenging^2^. Most of psychiatrists agree that early interventions may prove helpful in preventing the progression of the disease in patients^3,4^. Pharmacological treatment with psychosocial intervention is the current recommendation for individuals at high risk for BD^5^. However, there is some controversy over whether psychopharmacological intervention is needed in a high-risk state^6^. Concerns about over-medication or side effects of drugs make it difficult for psychiatrists to prescribe medications as a primary treatment^6,7^, although a few studies have demonstrated the efficacy of pharmacological intervention^8^. Therefore, if the biological pathophysiology observed in patients with BD is also altered in the prodromal stage, the strategy of early pharmacological intervention will be supported.

However, most biological alterations focused on BD patients with fully expressed mood episodes, such as hypomania, mania, or depression^9–13^. Few studies have been conducted on patients in high-risk states^14^. For example, the Dutch Bipolar Offspring study reported an aberrant neuroimmune state in bipolar offspring, compared to control offspring. These findings might reflect the vulnerability to BD, rather than a predictive marker^15^. Hayes, Khandaker^16^ reported that increased levels of interleukin-6 in childhood, which reflect a systemic inflammatory state, were associated with the onset of hypomanic symptom in adulthood. However, it is not possible to predict disease prognosis with a single marker; further useful markers are needed to predict disease prior to onset.

Therefore, this study identified whether protein markers are also altered in high-risk patients who do not meet the diagnostic criteria for BD. This study focused on recruiting subjects in a high risk state who were difficult to diagnose in terms of whether they were affected by depressive disorder or bipolar II disorder with respect to disease prognosis, and specifically, those who showed subthreshold hypomanic, depressive or cyclothymic symptoms but did not meet the diagnostic criteria at that time. Clinicians often encounter patients showing uncertain mood symptoms, which makes it difficult to prescribe therapeutic drugs based on their prognosis. Herein, blood proteins detected in high-risk subjects were compared to those in subjects with bipolar II disorder (BD-II) and a healthy control (HC) group. To recruit suitable subjects, we used the bipolar at-risk state (BARS) criteria, which were developed to identify individuals who are in a high-risk state for BDs^17^. According to Fusar-Poli, De Micheli^17^, those who met the BARS criteria exhibited a higher risk of developing BD than those who did not. Biochemical changes, which could reflect disease progression in BDs, have been reported via altered gene expression^18^, such as increased pro-inflammatory cytokines, oxidative stress, and cellular mitochondria dysfunction^19–21^. Some of these biochemical markers have been found to be associated with disease severity, regardless of the polarity of the subject’s mood state, and have recovered after remission or treatment^19^.

In addition, we used multiple reaction monitoring-mass spectrometry (MRM-MS) to simultaneously quantify several targeted peptides. MRM-MS can simultaneously measure hundreds of protein targets per sample with high sensitivity and accuracy, when compared to conventional technologies such as immunoassay, matrix-assisted laser desorption ionization-time-of-flight mass spectrometer (MALDI-TOF/TOF MS), and liquid chromatography-tandem mass spectrometry (LC–MS/MS). MRM-MS has recently been used in psychiatric research^22^, although few studies have applied this technique to high-risk groups. Various alterations of plasma proteins have been reported in psychiatric fields. Therefore, it is necessary to measure these variations simultaneously to ensure diagnostic accuracy. In this study, by applying this analytic technique, we aimed to identify changes in several blood protein markers that are characteristically observed in high-risk groups by comparing BD-II and HC patients.

## Results

### Demographic and clinical characteristics

Table 1 shows the demographic and clinical characteristics of the participants. The HC group included subjects with high education. The use of medication, antipsychotics and mood stabilizers, was more used in the patients in the BD-II group than in the BP group. The severity of manic symptoms (YMRS) was higher in the BD-II group than in the BP group. The other demographics (age and sex) and clinical characteristics (use of antidepressants, use of benzodiazepines, HAM-D, and CGI-S) were not significantly different between the groups. The classified subgroup of BP under BARS criteria is as follows: depression + cyclothymic features (n = 28, 70.0%), depression + genetic risk (n = 15, 37.5%), subthreshold mania (n = 5, 12.5%), mood swings (n = 1, 2.5%). No subjects met the subtype of cyclothymic features and genetic risk, subthreshold mixed episode. There were no significant differences between subgroups of BP in HAM-D, YMRS, and CGI-S (Table S2).

**Table 1:** Demographic and clinical characteristics of the study subjects.

Characteristics	HC (n = 75)	BP (n = 40)	BD-II (n = 48)	*p*-value^a^
Age, years (mean ± SD)	23.9 ± 4.1	22.1 ± 4.2	23.1 ± 4.7	0.088
Sex (Female), n (%)	38 (50.7)	27 (67.5)	31 (64.6)	0.147
Education, years (mean ± SD)	**15.0 ± 2.1**	**13.7 ± 2.3**	**13.9 ± 2.1**	**0.002**^**b**^
Age of symptom onset, years (mean ± SD)		16.4 ± 4.1	18.0 ± 5.0	0.112
Duration of untreated period, month (mean ± SD)		55.4 ± 54.6	46.73 ± 39.0	0.389
Psychopharmacology use, n (%)		25 (62.5)	39 (81.3)	0.058
Antipsychotics, n (%)		**18 (45.0)**	**34 (70.8)**	**0.014**
Mood stabilizers, n (%)		**15 (37.5)**	**37 (77.1)**	**< 0.001**
Antidepressants, n (%)		5 (12.5)	6 (12.5)	> 0.999
Benzodiazepines, n (%)		11 (27.5)	15 (31.3)	0.701
HAM-D Total (mean ± SD)		10.5 ± 6.4	11.4 ± 5.9	0.474
YMRS Total (mean ± SD)		**2.0 ± 2.4**	**3.5 ± 4.2**	**0.032**
CGI-S (mean ± SD)		3.3 ± 0.7	3.3 ± 0.7	0.957

Significant values are in bold.

^a^Significant differences between the healthy control, bipolar prodrome, bipolar II disorder samples were examined using the chi-square test for categorical variables, and analysis of variance (ANOVA) and independent samples t-test for continuous variables.

^b^HC showed longer duration of education when compared to BP and BD-II group.

*HC* Healthy control, *BP* Bipolar II prodrome, *BD-II* Bipolar II disorder, *SD* Standard deviation, *BMI* Body mass index, *HAM-D* Hamilton depression rating scale, *YMRS* Young mania rating scale, *CGI-S* Clinical global impression-severity scale.

### Comparison of proteins between HC, BP, and BD-II

Among the 143 target peptides (85 proteins), 26 peptides (24 proteins) were found to be significant after ANOVA with multiple comparisons (FDR < 0.05; Table S3). After adjusting covariates (age, sex, use of antipsychotics mood stabilizers, anti-depressants, and benzodiazepines) through ANCOVA, all peptides except peroxiredoxin 2 (PRDX2) were still significant (Table 2). Among these 25 peptides, 19 peptides were differentially expressed in the BP group (two peptides were upregulated and 17 peptides were downregulated in BPs, compared to the BD-II and HC groups) after multiple comparisons. Three peptides were significantly different when compared between the BP and BD-II groups (one peptide was upregulated and two were down regulated in the BP group versus the BD-II group). The other two peptides were downregulated in the BP group compared to the HC group.

**Table 2:** Differentially expressed peptides identified by LC-MRM analysis between HC, BP, and BD-II.

Protein ID	Gene name	Peptide	HC versus BP versus BD-II		BP versus BD-II		BP versus HC		BD-II versus HC	
			F-statistics	*p*-value (ANCOVA^a^)	Mean difference^b^	*p*-value (Tukey's HSD)	Mean difference	*p*-value (Tukey's HSD)	Mean difference	*p*-value (Tukey's HSD)
P10645	CHGA	ELQDLALQGAK	7.874	** < 0.001**	0.769	**0.001**	0.569	**0.018**	− 0.200	0.689
O00391	QSOX1	DTGAALLAESR	6.565	**0.002**	0.482	**0.003**	0.353	**0.036**	− 0.128	0.720
P67936	TPM4	TIDDLEEK	4.155	**0.018**	0.851	**0.013**	0.215	0.725	− 0.635	0.137
P04217	A1BG	SGLSTGWTQLSK	13.945	** < 0.001**	− 0.335	** < 0.001**	− 0.315	** < 0.001**	0.020	0.971
P04217	A1BG	ATWSGAVLAGR	11.503	** < 0.001**	− 0.335	** < 0.001**	− 0.297	** < 0.001**	0.038	0.914
P04275	VWF	VTVFPIGIGDR	6.091	**0.003**	− 0.609	**0.006**	− 0.481	**0.033**	0.128	0.843
P00742	F10	TGIVSGFGR	8.726	** < 0.001**	− 0.339	**0.001**	− 0.292	**0.005**	0.048	0.898
P01042	KNG1	QVVAGLNFR	7.823	** < 0.001**	− 0.277	**0.001**	− 0.213	**0.015**	0.064	0.754
P03952	KLKB1	TGAVSGHSLK	9.720	** < 0.001**	− 0.283	**0.002**	− 0.291	** < 0.001**	− 0.009	0.995
P03952	KLKB1	DSVTGTLPK	6.813	**0.001**	− 0.253	**0.042**	− 0.345	**0.002**	− 0.092	0.715
Q14624	ITIH4	FAHTVVTSR	8.298	** < 0.001**	− 0.271	**0.002**	− 0.235	**0.006**	0.036	0.914
O00187	MASP2	WPEPVFGR	5.970	**0.003**	− 0.263	**0.011**	− 0.234	**0.021**	0.029	0.955
P02652	APOA2	EQLTPLIK	9.481	** < 0.001**	− 0.300	**0.003**	− 0.324	** < 0.001**	− 0.024	0.969
O00533	CHL1	VIAVNEVGR	11.285	** < 0.001**	− 0.341	** < 0.001**	− 0.270	**0.002**	0.071	0.723
P23560	BDNF	QYFYETK	5.960	**0.003**	− 0.004	**0.008**	− 0.004	**0.028**	0.001	0.898
P06396	GSN	EGGQTAPASTR	11.576	** < 0.001**	− 0.385	** < 0.001**	− 0.297	**0.002**	0.088	0.662
P51884	LUM	ILGPLSYSK	13.055	** < 0.001**	− 0.346	** < 0.001**	− 0.307	** < 0.001**	0.039	0.905
P43251	BTD	LSSGLVTAALYGR	9.137	** < 0.001**	− 0.284	**0.002**	− 0.285	**0.001**	0.002	> 0.999
P02790	HEMO	QGHNSVFLIK	10.864	** < 0.001**	− 0.278	** < 0.001**	− 0.242	**0.001**	0.037	0.888
Q04756	HGFA	VANYVDWINDR	7.452	**0.001**	− 0.355	**0.003**	− 0.296	**0.012**	0.059	0.877
P61626	LYSC	WESGYNTR	8.701	** < 0.001**	− 0.352	** < 0.001**	− 0.175	0.094	0.177	0.169
P11226	MBL2	EEAFLGITDEK	4.417	**0.014**	− 0.501	**0.012**	− 0.260	0.270	0.242	0.437
P02647	APOA1	LLDNWDSVTSTFSK	5.026	**0.008**	− 0.166	0.087	− 0.220	**0.011**	− 0.055	0.810
P61769	B2M	VNHVTLSQPK	6.566	**0.002**	− 0.227	0.090	− 0.363	**0.002**	− 0.135	0.512
P06276	CHLE	FWTSFFPK	3.890	**0.022**	− 0.198	0.064	− 0.194	0.060	0.004	0.999
P32119	PRDX2	QITVNDLPVGR	2.002	0.139	− 0.204	0.551	0.239	0.418	0.443	0.117

Statistically significant differences were anlyzed by ANCOVA. Multiple comparison was performed by Tukey's HSD. Boldface *p*-values are significant *p*-value (< 0.05).

^a^Covariates : Age, sex, use of antipsychotics, mood stabilizers, antidepressants, and benzodiazepines.

^b^Adjusted mean difference.

*HC* healthy control, *BP* bipolar II prodrome, *BD-II* Bipolar II disorder, *ANCOVA* analysis of covariance; HSD, honestly significant difference.

### Relationship between DEPs and severity of symptoms

Two peptides, plasma kallikrein (KLKB1, r = 0.236, *p* < 0.05) and mannan-binding lectin serine protease 2 (MASP2, r = 0.228, *p* < 0.05) significantly showed positive correlations with YMRS scores and sulfhydryl oxidase 1 (QSOX1, r = -0.231, *p* < 0.05) showed negative correlations with YMRS (Table 3). There were no DEPs which correlated with HAM-D and CGI-S.

**Table 3:** Relationship between 26 peptides and demographic/clinical variables.

BP&BD-II (n = 88)	HAM-D		YMRS		CGI-S	
Protein_peptide sequence	Pearson r	*p*-value	Pearson r	*p-*value	Pearson r	*p-*value
CMGA_ELQDLALQGAK	− 0.026	0.810	− 0.206	0.054	0.042	0.697
QSOX1_DTGAALLAESR	0.052	0.630	− **0.231**	**0.031**	0.005	0.963
GELS_EGGQTAPASTR	− 0.077	0.476	− 0.034	0.753	− 0.084	0.437
LUM_ILGPLSYSK	− 0.070	0.515	0.122	0.259	− 0.124	0.249
A1BG_SGLSTGWTQLSK	0.089	0.407	0.100	0.353	0.007	0.950
A1BG_ATWSGAVLAGR	0.101	0.347	0.090	0.406	− 0.028	0.797
NCHL1_VIAVNEVGR	− 0.120	0.267	0.071	0.510	− 0.084	0.438
BTD_LSSGLVTAALYGR	0.058	0.593	0.037	0.734	0.021	0.846
APOA2_EQLTPLIK	− 0.015	0.893	0.051	0.640	− 0.035	0.747
KNG1_QVVAGLNFR	0.038	0.723	0.132	0.220	− 0.052	0.630
KLKB1_TGAVSGHSLK	0.094	0.386	**0.236**	**0.027**	0.047	0.665
KLKB1_DSVTGTLPK	0.065	0.545	0.208	0.052	0.020	0.850
HEMO_QGHNSVFLIK	0.125	0.247	0.183	0.087	0.030	0.781
FA10_TGIVSGFGR	0.175	0.104	0.094	0.385	0.142	0.188
ITIH4_FAHTVVTSR	− 0.086	0.424	0.078	0.469	− 0.126	0.242
HGFA_VANYVDWINDR	− 0.027	0.806	− 0.038	0.725	0.081	0.455
MASP2_WPEPVFGR	0.070	0.517	**0.228**	**0.032**	− 0.032	0.767
VWF_VTVFPIGIGDR	− 0.028	0.793	0.039	0.719	− 0.046	0.668
BDNF_QYFYETK	− 0.108	0.317	0.149	0.165	− 0.114	0.288
CHLE_FWTSFFPK	0.169	0.116	0.176	0.100	0.008	0.942
TPM4_TIDDLEEK	0.091	0.408	− 0.020	0.857	0.012	0.915
LYSC_WESGYNTR	− 0.053	0.626	0.198	0.064	− 0.035	0.744
MBL2_EEAFLGITDEK	− 0.152	0.156	0.046	0.668	− 0.049	0.648
APOA1_LLDNWDSVTSTFSK	− 0.106	0.328	− 0.090	0.406	− 0.125	0.245
B2MG_VNHVTLSQPK	0.143	0.184	0.095	0.378	0.073	0.500
PRDX2_QITVNDLPVGR	0.070	0.520	0.058	0.593	0.063	0.559

Significant values are in bold.

*BP* bipolar II prodrome, *BD-II* Bipolar II disorder, *HAM-D* Hamilton depression rating scale, *YMRS* Young mania rating scale, *CGI-S* Clinical global impression-severity scale.

### Bioinformatics

**Figure 1:**
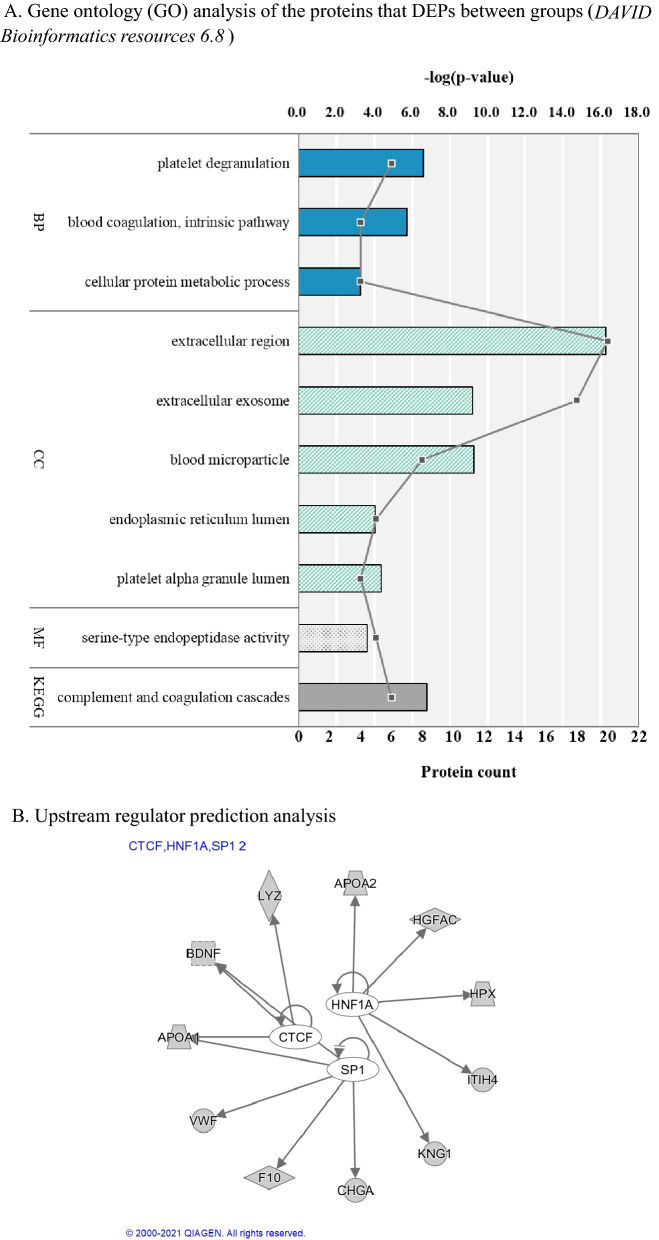
Bioinformatics analysis of the differentially expressed proteins.

Twenty-four DEPs were included in bioinformatics analysis. Most proteins were involved in the complement and coagulation cascades, and to a lesser extent, the cellular protein metabolic process. Cellular component analysis results showed that most protein markers were related to the composition of extracellular space and to the endoplasmic reticulum. Molecular function analyses showed that markers were related to serine-type endopeptidase (Fig. 1A). To identify the cascade of transcriptional regulator for the 24 DEPs, we constructed a regulator-target network using upstream regulator prediction analysis (Fig. 1B). As a result, three upstream regulators (CTCF, HNF1A, and SP1) that regulate 24 DEPs were discovered.

## Discussion

Most of the peptides that showed significant results in this study showed decreased or increased expression in BPs than in other groups. Altered expression of these findings have been reported through extensive studies related to psychiatric disorders, including BD^23–25^. Bioinformatics analysis showed that Sp1, CCCTC-binding factor (CTCF), and hepatocyte nuclear factor 1-alpha-A (HNFA1) were upstream regulators of these significant proteins. Sp1 is a transcription factor related to vulnerability to BDs^26^, and CTCF is a regulator of learning genes^27^. According to Moos et al.^28^, alterations in specific areas of the brain related to mental illness can also be detected in blood. In particular, proteins secreted from neurons and neurons can be detected in the blood, suggesting that abnormalities in the central nervous system can be identified in peripheral blood^28^. Some proteins were reduced with BP in this study, such as APOA1, APOA2, BDNF, and GSN, which were also reported to show decreased expression in cerebrospinal fluid (CSF). However, the expression of HPX, which showed decreased expression with BP in this study, was shown to be increased, and A1BG showed an inconsistent pattern of alterations in CSF. Further validation studies will be needed to confirm these inconsistent alterations in proteins between peripheral blood and CSF. Our findings in the high-risk group suggest that these pathophysiological changes might start in the early stages of BD. In addition, the results suggest that these changes present more actively in patients in the prodromal stage than in patients with fully expressed mood episodes.

Two proteins, chromatogranin-A (CHGA) and QSOX1, exhibited overexpression in the BP group. The overexpression of CHGA has been reported in schizophrenia and BDs^29^, and its function has been related to pro-inflammation and impaired neurotransmission^30^. Although QSOX1 has not been studied in relation to mental disorders, it has been linked to lower survival in patients with breast cancer through disturbing the proliferation cycle^31^. Therefore, these proteins might relate to disease progression, such as neuroinflammation, and could be targets for the treatment of BDs.

Proteins that were under-expressed in the BP group, were related to blood coagulation (A1BG, Alpha-1B-glycoprotein; vWF, von Willebrand factor; F10, Coagulation factor X; KNG1, Kininogen-1; KLKB1, Plasma kallikrein), immune response (ITIH4, Inter-alpha-trypsin inhibitor heavy chain H4; MASP2, mannan-binding lectin-associated serine protease-2; MBL2, Mannan-binding lectin serine protease 2; LYSC, Lysozyme C; B2M, Beta-2-microglobulin), lipid metabolism (APOA1, Apolipoprotein A-I; APOA2, Apolipoprotein A-II), and the development of synaptic plasticity (CHL1, Neural cell adhesion molecule L1-like protein; BDNF, Brain-derived neurotrophic factor). Mental stress can activate a blood coagulation cascade^32,33^ in response to catecholamine and serotonin stimuli. A1BG, KNG1, and KLKB1 have also been reported to be under-expressed in BP-II patients^34^. In addition, previous studies have reported decreased expression of the anti-inflammatory protein, ITIH4, and of proteins related to innate immunity (MASP2 and MBL2) in BDs, suggesting increased inflammatory reactions and vulnerable state to infection^10,25^. Early perturbations in cholesterol metabolism have also been reported in psychiatric disorders, and lower serum cholesterol could relate to dysfunctions regarding the development of myelin and synaptic formation^25^. CHL1 and BDNF, which are proteins related to the synaptic plasticity of neurons^35,36^, exhibited reduced expression in BP patients. In addition to these pathological pathways, various neuro-protective proteins, such as gelsolin (GSN), which prevents apoptosis through calcium regulation^37^; cholinesterase (CHLE), which can degrade neurotoxic substrates; and hemopexin (HPX) and hepatocyte growth factor activator (HGFA), which are associated with oxidative stress reduction, have also been reported in prior psychiatric studies^25,38,39^. These proteins with decreased levels were related to the dysfunction of the prevention of disease progression, acute immune reaction, neuro-protective function, and neuronal plasticity. Therefore, our findings support the theory that the dysfunctions of mechanisms can be observed in the early stage of the disease. In addition, consistent with previous reports^35,38^, these alterations may have been normalized in patients with BD-II who had already started treatment after complete expression of their symptoms.

Peroxiredoxin-2 (PRDX2) was overexpressed throughout the disease progression (HC < BP < BD-II) in ANOVA analysis. The results were no more significant after adjusting for medication usage. However, the overexpression of PRDX2 has been reported in prior studies^11,12^; further longitudinal research is needed to identify this association with the disease’s progression.

In contrast to previous findings, which compared healthy control patients and BD patients^12^, no differentiation was detected between BD-II and HC, except for PRDX2. This is likely because only three target peptides (PRDX2, F13A, and GAPDH) which were identified as being significant by Kim et al. (2021) were included in this study (due to the stability and measurability of the target materials). Moreover, BD-specific markers that have been reported in prior studies were not found to be significant in this study^23–25^. These results might reflect the treatment state; most patients with BD-II used their medication, and BP patients were excluded if they had taken antipsychotics for more than three weeks, or if they had taken mood stabilizers for more than six weeks. Previous studies have reported that markers can be recovered after treatment. Therefore, our results might reflect this phenomenon^19,20,35,40^.

Although this study is robust in that blood proteins were directly measured in BP patients, there are a few limitations. First, we used cross-sectionally recruited samples in this analysis, and therefore, our findings might be insufficient to prove the causality of disease progression. Further analysis using samples collected during follow ups with the BP patients will be conducted in the future. Second, although we statistically corrected confounding factors that could affect protein expression (covariates as age, sex, and drug use), we did not adjust for variables, such as body mass index, smoking habit, fasting state, duration/dosage of medication. However, we conducted statistical efforts to eliminate the effects of drug use, and we have confirmed that the use of medication did not change the results significantly. In addition, there was no DEP between the drug use patients group and drug free patients group (Table S4). These results, however, cannot exclude drug effects on protein expression, and further research reflecting drug dosage and duration of use will be needed in the future. In this study, the effect of disease progression on protein expression might have been greater than that of drug administration. Third, though differentiation between the prodromal stage of bipolar I and bipolar II is unclear, our study focused on the recruitment of patients in the prodromal stage of bipolar II (75% of BP met “depression + cyclothymic features” criteria). The blood proteins of the BP group were only compared with those of BD-II, except BD-I. Nevertheless, we also included patients with BP with depression + genetic-risk bipolar disorder (either BD-I or BD-II; n = 15, 37.5%) and subthreshold mania (n = 5, 12.5%). Therefore, it is necessary to compare the expression of protein markers in patients with BP with BD-I in a further study. A further study will extend the definition of a high risk state of BD, with important implications for finding protein markers that reflect high-risk conditions for overall bipolar disorders. Fourth, since the aim of this study was the discovery of protein markers related to a high-risk state with bipolar disorders, validation with other technologies such as immunoassays was not performed. However, the LC-MRM-MS method has several advantages such as ELISA-level sensitivity and reproducibility and good multiplexing capability^41^. In the case of mental disorders, it is difficult to accurately diagnose or predict prognosis with a single protein marker or a small number of proteins^42,43^. For clinical applications in the psychiatric field, it might be helpful to develop a multi-marker panel that combines expression patterns of multiple proteins. Several studies have been reported in which only MRM-MS analysis was used to discover, develop, and clinically apply protein markers^41,44,45^. Therefore, to quantify the 24 blood protein candidates found in this study in more than 100 blood samples, considering cost and clinical applicability, it is technically advantageous to use MRM-MS analysis rather than immunoassay analysis in future validation studies. Fifth, this study is to discover protein markers associated with high risk groups with bipolar disorder, replicate our results, an independent validation set will be needed in future studies.

Despite these limitations, by comparing the protein expressions of the BP group with the BD-II and HC groups, it was possible to compare our findings with previously reported results for patients with BD-II. In particular, our results were meaningful in that we confirmed that pathological abnormalities (blood coagulation, immune response, lipid metabolism, and neuronal plasticity) that have been consistently reported in previous studies in patients could also be observed in high-risk state of BDs. Therefore, in future studies, it seems necessary to further explore pathological abnormalities in connection with genetic or environmental factors related to pathological abnormalities commonly observed in mental illness. In addition, a longitudinal study will be needed to confirm whether these pathological abnormalities are normalized after medication intervention.

## Methods

### Participants

We recruited 40 patients with prodromal bipolar II disorder (BP), 48 patients with BD-II, and 75 patients for the HC group. The age of all participants was limited to 15–35 years, which is known to be the most frequent age of BD onset^46^. BP patients who had distressful mood symptoms but did not meet the diagnostic criteria of BD-II and major depressive disorder were referred from five institutions (Seoul National University Hospital; SMG-SNU Boramae Medical Center, Hanyang; University Hospital; National Mental Health Center; and Gwanakgu Public Health Center) in Seoul, South Korea. All participants attended the Seoul Youth Clinic or the Mood disorders Clinic of Seoul National University Hospital, where they were evaluated by experienced psychiatrists and trained psychiatric specialists. The BARS criteria were used in this study to recruit prodromal-stage BD-II patients^17^. According to BARS criteria, the following six inclusion criteria were used to define the BP over the last 12 months: (1) subthreshold mania, (2) depression + cyclothymic features, (3) depression + genetic risk, (4) cyclothymic features and genetic risk, (5) subthreshold mixed episode, and (6) mood swings. All details of the inclusion criteria for the BP group have been described in Fusar-Poli et al. (2018) and in a previously published article^47^. The exclusion criteria of BP were as follows: (1) history of psychosis that lasted more than seven days regardless of treatment, (2) history of mood stabilizer treatment for six weeks or more, (3) history of antipsychotic treatment for more than three weeks, and (4) history of bipolar I or II disorder.

Patients who had been diagnosed with BD-II either by the Diagnostic and Statistical Manual of Mental Disorders (DSM)-IV or by DSM-5 diagnostic criteria within the previous five years were recruited from the Seoul National University Hospital. The age- and sex-matched HC patients did not have any history of psychiatric disorders and had no family history of psychiatric disorders within second-degree relatives. All participants who had a prior history of receiving neuromodulation therapy, neurosurgery, any central nervous system disease (including epilepsy), severe head trauma with loss of consciousness, thyroid disease, mental retardation, or substance abuse were excluded.

Written informed consent was obtained from all subjects before interview. For participants under the age of 18 years, informed consent was obtained from both the participant and their parents/guardians. The study protocol was approved by the Institutional Review Board of the Seoul National University Hospital (IRB No. 1704-075-846) and was performed in accordance with the tenets of the Declaration of Helsinki.

### Clinical assessments

All participants were assessed by the structured clinical interview for DSM-IV-TR axis I disorders (SCID-I)^48^ or by the Mini-International Neuropsychiatric Interview (MINI). SCID -I and MINI are semi-structured interviews for diagnosing major psychiatric disorders^48^. In this study, these tools were used to confirm that BP did not meet the diagnostic criteria for bipolar disorders and to diagnose patient with BD-II.

The Hamilton Depression Rating Scale (HAM-D)^49,50^, the Young mania rating scale (YMRS)^51^, and the clinical global impression-severity scale (CGI-S)^52^ were used to assess the severity of symptoms in participants with BP and BD-II. The HAM-D included 17 items for assessing depressive symptoms and the range of the total score is 0–52^49,50^. The YMRS measures mania symptoms with 11 items, and the range of the total score is 0–60. The CGI-S was used to assess their global symptom severity ranging from 1 (not ill) to 7 (extremely severe). All assessments were rated by an interviewer, and a higher score indicated more severe symptoms. Symptom evaluations and blood sampling were performed on the same day.

### Plasma sample preparation and protein quantification by MRM-MS

Ethylenediaminetetraacetic acid (EDTA) tubes were used to collect blood samples. The plasma samples were centrifuged at 3000 rpm at 4 °C for 10 min. The samples were then stored at below − 70 °C until further analysis.

Clinical plasma samples were proteolytically digested with trypsin, according to a previously-described one-step digestion process^12,53,54^. Briefly, digestion buffer (8 M urea, 5 mM TCEP, 20 mM CAA in 0.1 M ABC) was added to 2 µl of plasma sample. The mixture was boiled for 30 min at 60 °C to denature and alkylate the proteins. After cooling to room temperature, protein digestion was performed at 37 °C overnight, using trypsin at a 75:1 protein-to-protease ratio. All resulting peptides were acidified with 10% trifluoroacetic acid and desalted using homemade C18-StageTips as described^55^. Desalted samples were completely dried with a vacuum dryer and stored at − 80 °C.

Desalted peptide samples were spiked with crude stable isotope-labeled internal standard (SIS) peptide, with a C-terminal lysine or arginine heavy-isotope-labeled (13C6 15N2 or 13C6 15N4) [purity: crude (> 70%), JPT, Berlin, Germany] and iRT peptides (Biognosys AG, CH) as internal standards. The samples were randomly distributed in blocked batches and labeled with identification numbers to blind the researchers throughout the sample preparation.

The LC-MRM-MS platform consisted of a 1290 Infinity ultra-high performance liquid chromatography system interfaced to a 6495 triple quadrupole mass spectrometer via a Jet Stream ESI source (Agilent Technologies; Santa Clara, CA, USA). Solvents A and B for the UHPLC consisted of 0.1% formic acid/water (v/v) and 0.1% formic acid/acetonitrile (v/v), respectively. The gradient was set up to start with a 2% organic mobile phase, increased to 30% at 32 min, 45% at 35 min, 90% at 35.5 min, and remained at 90% until 37.5 min, before returning to 2% at 38 min and then remaining at 2% until 40 min. A post-gradient column re-equilibration of 4 min was used after the analysis of each plasma sample and blank.

MS analysis was performed on an Agilent 6495A triple quadrupole instrument operated in the positive ion mode. For this study, 92 proteins, corresponding to 158 peptides and 474 transitions, were included for targeted MRM-MS. These proteins and the corresponding peptides are summarized in Supplementary Table S1. Approximately 20 µg of digested peptides was injected per LC-MRM-MS run. MRM data were acquired at a 3000 V capillary voltage and a 500 V nozzle voltage. The sheath gas flow was set to 12 L min^−1^ at a temperature of 350 °C, and the drying gas flow was set to 15 L min^−1^ at a temperature of 250 °C; the nebulizer gas pressure was set to 25 psi. The collision cell accelerator voltage was set to 5 V, and unit mass resolution was used in the first and third quadrupole mass analyzers. Collision energy was optimized by adding the intensities of individual transitions that resulted in the largest peak area. The high energy dynode multiplier was set to − 20 kV to improve the ion detection efficiency and signal-to-noise ratios. The top three transitions per peptide target were monitored over 900 ms cycles, and 60 s detection windows were used for quantitative analysis.

### MRM-MS data processing

Initially, the raw data from the MRM-MS analysis were processed in Skyline v. 20.2 (MacCoss Lab, Seattle, WA, USA) to calculate the peak area values of the transitions. Peptide quantification was conducted based on the relative abundance of the endogenous and SIS peptide transitions, that is, the relative abundance of the transition pair (Q1 and Q3) was determined using the ratio of endogenous (light) peptide peak areas to the SIS (heavy) peptide peak areas. These data were reported as peak area ratios (PAR) and light/heavy (L/H) ratios.

### Statistical analyses

As we collected and stored patients’ blood samples from two different clinics (the Seoul Youth Clinic and Mood disorders Clinic) in Seoul National University Hospital, we used Combat algorithms (http://genepattern.boadinstitute.org) to reduce batch effects^56^. After this correction, 143 peptides (85 proteins) were selected for statistical analysis (Table S1, Figure S1); 15 other peptides (7 proteins) were excluded as batch effects were still observed. Subsequently, a log2 transformation of the values was conducted due to the skewed distribution of the data. The normality of the protein data was assessed using the Shapiro–Wilk test.

Furthermore, the chi-square test and Fisher’s exact test were conducted for categorical data, and independent samples t-tests, Mann–Whitney U-tests, Kruskal–Wallis H test, and analysis of variance (ANOVA) were conducted for continuous variables, including proteomic data. As the expression of proteins is affected by patients’ age^57^, sex^58^, and the use of psychotropic drugs (antipsychotics, mood stabilizers, antidepressants, benzodiazepines)^59^, analysis of covariance (ANCOVA) with multiple comparison (Tukey's HSD) was performed on differently expressed proteins (DEPs) that were significant in ANOVA to control the effects of covariates. Correlation analysis was conducted to evaluate the relationships between clinical symptoms (HAM-D total, YMRS total, and CGI-S). Multiple protein markers (143 peptides, 85 proteins) were included for statistical analysis, and *p*-values were adjusted for multiple comparisons using the Benjamini–Hochberg false discovery rate (FDR). FDR values < 0.05 were considered to signify DEPs between each group. Bioinformatics analysis was conducted using the Database for Annotation, Visualization and Integrated Discovery (DAVID) v. 6.8 (http://david.ncifcrf.gov/) to obtain the Gene Ontology (GO) terms^60^. Upstream regulator prediction was performed by Ingenuity Pathway Analysis (IPA, QIAZEN, Hilden, Germany) based on the 24 DEPs. Statistical analyses were performed using IBM SPSS 25.0. (IBM Inc., Armonk, NY, USA).

### Ethics approval and consent to participate

Written informed consent was obtained from all patients before participation in the study. In case of patients below 18 years of age, informed consent was obtained from both the patients and their parents/guardians. The study protocol was approved by the Institutional Review Board of Seoul National University Hospital (IRB No. 1704-075-846) and was conducted in accordance with the principles of Declaration of Helsinki.

## Conclusion

In conclusion, this study identified 24 plasma proteins that could reflect disease progression for BDs. The most significant protein alterations were observed in the BP group, suggesting that the alterations of biological pathways could start in the high-risk state. In particular, these alterations in the early stage might be more related to a weakened protective function resulting from disease progression than to disease progression. These results suggest that the pathological mechanisms might start before the symptoms are fully expressed, and therefore early intervention with drug use might be needed. Longitudinal studies are needed to identify whether the alterations of the proteins could be useful as predictive markers for the disease progression of BDs.

## Supplementary Information

Supplementary Figure S1.

Supplementary Tables.

Supplementary Legends.

## Data Availability

Data sharing is not applicable to this article as no datasets were generated or analyzed during the current study.
