# Supplementary material for: Alterations in blood proteins in the prodromal stage of bipolar II disorders
Source: Sci Rep. 2022 Feb 24;12:3174. doi: 10.1038/s41598-022-07160-0 (PMC8873249; doi:10.1038/s41598-022-07160-0)
Supplement: Supplementary file 1 — Supplementary Figure S1. [file 41598_2022_7160_MOESM1_ESM.pptx]

## Slide 1
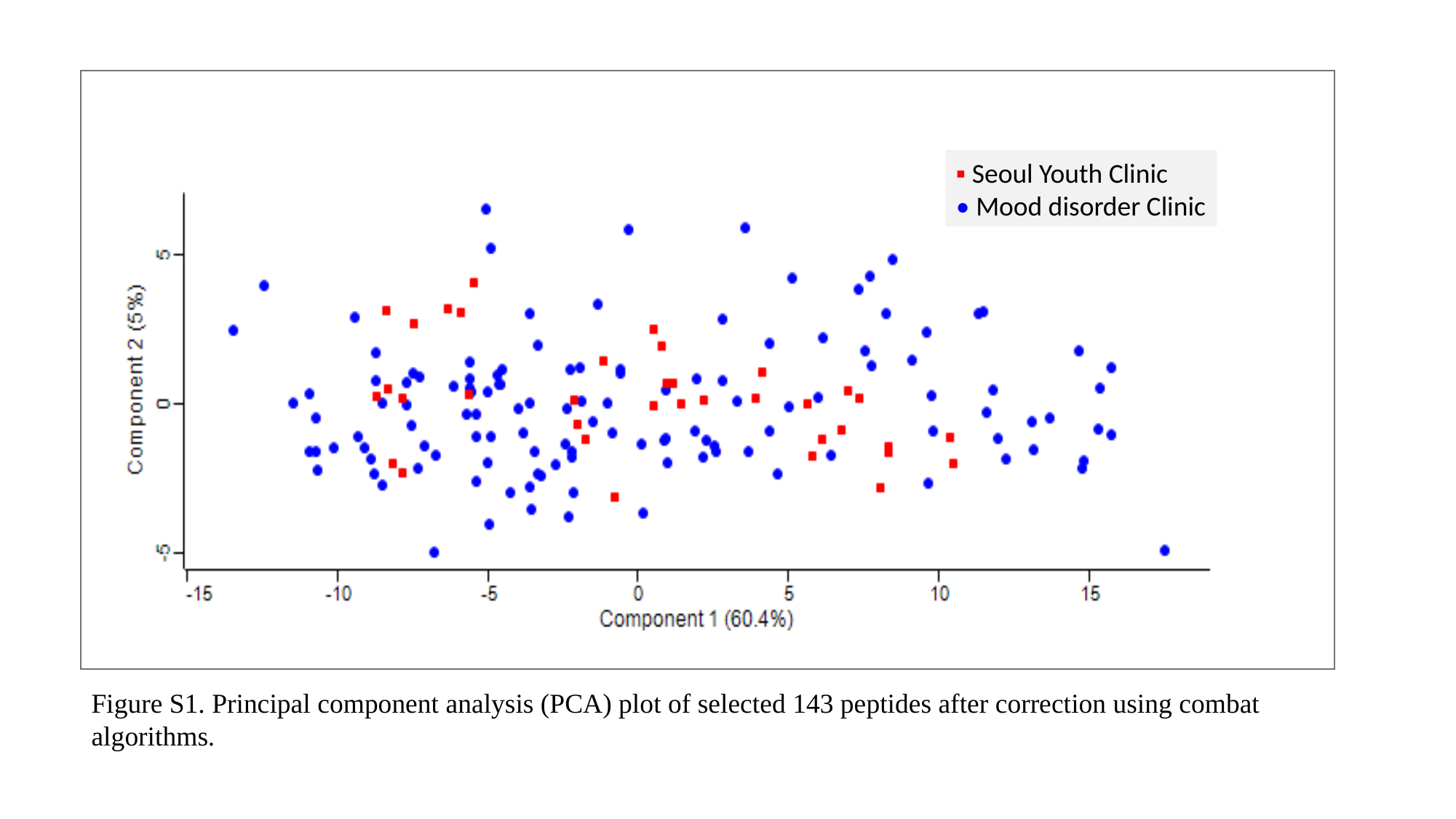

▪ Seoul Youth Clinic
• Mood disorder Clinic
Figure S1. Principal component analysis (PCA) plot of selected 143 peptides after correction using combat algorithms.
