# Supplementary material for: Alterations in blood proteins in the prodromal stage of bipolar II disorders
Source: Sci Rep. 2022 Feb 24;12:3174. doi: 10.1038/s41598-022-07160-0 (PMC8873249; doi:10.1038/s41598-022-07160-0)
Supplement: Supplementary file 3 — Supplementary Legends. [file 41598_2022_7160_MOESM3_ESM.docx]

**(Supplementary Information)**

**Supplement Tables**

Table S1.xlsx: The 143 peptides (85 proteins) examined by MRM-MS. UniProt accession number, protein ID, and information pertaining to mass spectra are listed for each peptide.

Table S2.xlsx: Classification of subgroup of bipolar II prodrome according to BARS criteria

Table S3.xlsx: Differentially expressed peptides identified by LC-MRM analysis between HC, BP, and BD-II.

Table S4.xlsx: Effect of drug use on differentially expressed peptides identified by LC-MRM analysis

A. Comparison between drug use patients (n=64) and drug free patients (n=24).

B. Comparison between drug use BP (n=25) and drug free BP (n=15)

**Supplement Figures**

Figure S1.pptx: Principal component analysis (PCA) plot of selected 143 peptides after correction using combat algorithms.
